# Supplementary material for: Functional trade-offs and environmental variation shaped ancient trajectories in the evolution of dim-light vision
Source: eLife. 2018 Oct 26;7:e35957. doi: 10.7554/eLife.35957 (PMC6203435; doi:10.7554/eLife.35957)
Supplement: Supplementary file 1. [file elife-35957-supp1.docx]

**Supplementary File 1**

| **Species** | ***Rh1* Accession Number** |
| --- | --- |
| *Alligator mississippiensis* | NM_001287282.1 |
| *Alligator sinensis* | XM_006039400.1 |
| *Ambystoma tigrinum* | U36574.1 |
| *Anas platyrhynchos* | XM_005012054.3 |
| *Anolis carolinensis* | NM_001291387.1 |
| *Balaenoptera acutorostrata scammoni* | XM_007192608.1 |
| *Bos indicus* | XM_019984265.1 |
| *Bos mutus* | XM_005902834.1 |
| *Bubalus bubalis* | XM_006078900.1 |
| *Callithrix jacchus* | XM_008982215.2 |
| *Camelus ferus* | XM_006180073.1 |
| *Canis lupus familiaris* | XM_005632035.2 |
| *Cavia porcellus* | NM_001173085.1 |
| *Ceratotherium simum simum* | XM_004442424.2 |
| *Chelonia mydas* | XM_007059947.1 |
| *Chinchilla lanigera* | XM_005387085.2 |
| *Chlorocebus sabaeus* | XM_007985378.1 |
| *Chrysemys picta bellii* | XM_008168043.1 |
| *Chrysochloris asiatica* | XM_006868670.1 |
| *Condylura cristata* | XM_004692360.2 |
| *Cricetulus griseus* | NM_001244407.1 |
| *Cynops pyrrhogaster* | AB043890.1 |
| *Dasypus novemcinctus* | XM_004477246.1 |
| *Echinops telfairi* | XM_004702378.1 |
| *Elephantulus edwardii* | XM_006901040.1 |
| *Eptesicus fuscus* | XM_008152292.1 |
| *Equus caballus* | XM_001490301.4 |
| *Erinaceus europaeus* | XM_007517079.1 |
| *Falco cherrug* | XM_005443603.2 |
| *Felis catus* | NM_001009242.1 |
| *Ficedula albicollis* | XM_005053322.1 |
| *Gallus gallus* | NM_001030606.1 |
| *Geospiza fortis* | XM_005426641.1 |
| *Gorilla gorilla gorilla* | XM_004036292.2 |
| *Heterocephalus glaber* | XM_004870461.2 |
| *Homo sapiens* | AB464154.1 |
| *Ictidomys tridecemlineatus* | XM_005333784.3 |
| *Jaculus jaculus* | XM_004651581.1 |
| *Latimeria chalumnae* | XM_005997817.2 |
| *Leptosomus discolor* | XM_009949111.1 |
| *Lipotes vexillifer* | XM_007461564.1 |
| *Loxodonta africana* | NM_001280858.1 |
| *Macaca mulatta* | XM_001094250.2 |
| *Meleagris gallopavo* | XM_003210211.3 |
| *Melopsittacus undulatus* | AF021242.1 |
| *Mesocricetus auratus* | XM_021224801.1 |
| *Microtus ochrogaster* | XM_005365058.2 |
| *Monodelphis domestica* | XM_001366188.2 |
| *Mus musculus* | XM_017321493.1 |
| *Mustela putorius furo* | XM_004738577.1 |
| *Myotis brandtii* | XM_005870029.2 |
| *Myotis davidii* | XM_006758300.2 |
| *Myotis lucifugus* | XM_006083811.2 |
| *Neoceratodus forsteri* | EF526295.1 |
| *Nomascus leucogenys* | XM_003265030.3 |
| *Ochotona princeps* | XM_004581320.1 |
| *Octodon degus* | XM_004645617.2 |
| *Odobenus rosmarus divergens* | XM_004395657.1 |
| *Orcinus orca* | XM_004284305.2 |
| *Ornithorhynchus anatinus* | NM_001127627.1 |
| *Orycteropus afer afer* | XM_007956743.1 |
| *Oryctolagus cuniculus* | NM_001082349.1 |
| *Ovis aries* | XM_004018534.3 |
| *Pan paniscus* | XM_003829435.3 |
| *Pan troglodytes* | XM_516740.6 |
| *Pantholops hodgsonii* | XM_005955745.1 |
| *Papio anubis* | XM_003906878.3 |
| *Pelodiscus sinensis* | XM_006132837.2 |
| *Peromyscus maniculatus bairdii* | XM_006978532.1 |
| *Physeter catodon* | XM_007126220.1 |
| *Pongo abelii* | XM_002813145.3 |
| *Pseudopodoces humilis* | XM_005521947.1 |
| *Pteropus alecto* | XM_006917646.1 |
| *Python bivittatus* | XM_007423262.1 |
| *R.norvegicus* | NM_033441.1 |
| *Saimiri boliviensis boliviensis* | XM_003926159.2 |
| *Sarcophilus harrisii* | XM_003762449.1 |
| *Sorex araneus* | XM_004613232.1 |
| *Sus scrofa* | NM_214221.1 |
| *Taeniopygia guttata* | NM_001076695.1 |
| *Tarsius syrichta* | XM_008047820.2 |
| *Trichechus manatus latirostris* | XM_004368355.1 |
| *Tupaia chinensis* | XM_006160664.1 |
| *Tursiops truncatus* | NM_001280659.1 |
| *Vicugna pacos* | XM_006206787.1 |
| *Xenopus laevis* | NM_001087048.2 |
| *Xenopus tropicalis* | NM_001097334.2 |
| *Zonotrichia albicollis* | XM_005490154.1 |
