# Supplementary material for: Functional trade-offs and environmental variation shaped ancient trajectories in the evolution of dim-light vision
Source: eLife. 2018 Oct 26;7:e35957. doi: 10.7554/eLife.35957 (PMC6203435; doi:10.7554/eLife.35957)
Supplement: Supplementary file 2. [file elife-35957-supp2.docx]

**Supplementary File 2.**

| **Organism** | **Accession Number** |
| --- | --- |
| *Acanthopagrus berda* | JQ638362.1 |
| *Acanthopagrus butcheri* | DQ354577.1 |
| *Acanthopagrus schlegelii* | JQ638368.1 |
| *Alepocephalus bicolor* | JN230974.1 |
| *Anableps anableps* | EU637935.1 |
| *Anchoa colonensis* | KT201140.1 |
| *Anchovia surinamensis* | KT201133.1 |
| *Antennarius striatus* | KC442240.1 |
| *Aphanopus carbo* | EU637938.1 |
| *Aplodactylus punctatus* | EU637939.1 |
| *Arapaima gigas* | JN230972.1 |
| *Argentina sialis* | JN230995.1 |
| *Argyropelecus aculeatus* | JN412571.1 |
| *Ateleopus japonicus* | KC442218.1 |
| *Atractosteus tropicus* | JN230970.1 |
| *Balistes capriscus* | KC442242.1 |
| *Bathysaurus mollis* | JN412586.1 |
| *Bathytroctes microlepis* | JN544540.1 |
| *Benthosema suborbitale* | JN412576.1 |
| *Callionymus schaapii* | EU637946.1 |
| *Callorhinchus milii* | NM_001292252.1 |
| *Cataetyx laticeps* | EU637947.1 |
| *Channa maculata* | KF017146.1 |
| *Chanos chanos* | JN230981.1 |
| *Cheilodactylus quadricornis* | KF017159.1 |
| *Cheilopogon heterurus* | EU637950.1 |
| *Chlorophthalmus acutifrons* | KC442222.1 |
| *Conocara salmoneum* | JN412577.1 |
| *Coreoleuciscus splendidus* | JN003301.1 |
| *Coryphaenoides guentheri* | JN412578.1 |
| *Cubiceps gracilis* | EU637952.1 |
| *Dactyloptena orientalis* | KC442232.1 |
| *Diodon holocanthus* | KC442241.1 |
| *Diplomystes nahuelbutaensis* | JN230990.1 |
| *Distichodus antonii* | JN230985.1 |
| *Echiodon cryomargarites* | EU637956.1 |
| *Eigenmannia virescens* | KX260614.1 |
| *Elassoma zonatum* | EU637957.1 |
| *Epigonus telescopus* | EU637959.1 |
| *Esox americanus* | JN230998.1 |
| *Galaxias maculatus* | JN231000.1 |
| *Gasterosteus aculeatus* | EU637962.1 |
| *Gomphosus varius* | KP881294.1 |
| *Grammicolepis brachiusculus* | EU637964.1 |
| *Gymnothorax tile* | KY026033.1 |
| *Gymnotus tigre* | KY026038.1 |
| *Halosauropsis macrochir* | JN544541.1 |
| *Hepsetus odoe* | JX470079.1 |
| *Himantolophus groenlandicus* | EU637965.1 |
| *Hippoglossus stenolepis* | KF312141.1 |
| *Holocentrus rufus* | KC442230.1 |
| *Hoplostethus mediterraneus* | JN412583.1 |
| *Howella brodiei* | EU637966.1 |
| *Ichthyococcus ovatus* | JN412569.1 |
| *Indostomus paradoxus* | EU637967.1 |
| *Labrus bergylta* | KC442239.1 |
| *Lagocephalus lagocephalus* | EU637968.1 |
| *Lampanyctus alatus* | JN412575.1 |
| *Lampris guttatus* | KC442226.1 |
| *Lamprogrammus shcherbachevi* | EU637969.1 |
| *Lepisosteus oculatus* | JN230969.1 |
| *Lipophrys pholis* | HM630123.1 |
| *Liza aurata* | KF017144.1 |
| *Lophiodes iwamotoi* | KF060342.1 |
| *Lycengraulis grossidens* | KT201146.1 |
| *Malapterurus microstoma* | JX470084.1 |
| *Melamphaes suborbitalis* | JN231006.1 |
| *Melanochromis auratus* | AY775115.1 |
| *Melanotaenia australis* | FJ940704.1 |
| *Menidia menidia* | EU637977.1 |
| *Merluccius merluccius* | JN231004.1 |
| *Microcanthus strigatus* | EU637978.1 |
| *Mormyrops anguilloides* | JN230973.1 |
| *Mullus surmuletus* | EU637982.1 |
| *Mylochromis lateristriga* | AY775119.1 |
| *Myripristis murdjan* | KC442231.1 |
| *Nandus nebulosus* | KF017145.1 |
| *Naso lituratus* | EU637984.1 |
| *Nemadactylus monodactylus* | EU637985.1 |
| *Neoscopelus microchir* | KC442224.1 |
| *Oncorhynchus gorbuscha* | AY214151.1 |
| *Oncorhynchus tshawytscha* | AY214136.1 |
| *Ophiocara porocephala* | EU637988.1 |
| *Opsanus tau* | KC442229.1 |
| *Orectolobus ornatus* | JX534164.1 |
| *Oreochromis niloticus* | AY775108.1 |
| *Osmerus eperlanus* | JN230996.1 |
| *Osteoglossum bicirrhosum* | KY026030.1 |
| *Pagetopsis macropterus* | EU637990.1 |
| *Pagrus major* | JQ638374.1 |
| *Parakneria cameronensis* | JN230982.1 |
| *Paralichthys dentatus* | KU980166.1 |
| *Pelates quadrilineatus* | EU637991.1 |
| *Percopsis omiscomaycus* | KC442228.1 |
| *Photoblepharon palpebratum* | EU637993.1 |
| *Phycis phycis* | EU637994.1 |
| *Pinguipes chilensis* | EU637989.1 |
| *Poecilia reticulata* | NM_001312652.1 |
| *Polymixia japonica* | JN231005.1 |
| *Polymixia lowei* | KC442227.1 |
| *Prionurus scalprum* | KC442238.1 |
| *Pterygoplichthys sp. JM-2016* | KY026039.1 |
| *Romanogobio ciscaucasicus* | JN003308.1 |
| *Schedophilus medusophagus* | EU638003.1 |
| *Scophthalmus rhombus* | EU638005.1 |
| *Sebastes umbrosus* | EF212438.1 |
| *Semaprochilodus insignis* | JX470089.1 |
| *Sewellia lineolata* | KY026034.1 |
| *Sphaeramia nematoptera* | EU638010.1 |
| *Steatogenys elegans* | JX470090.1 |
| *Synagrops japonicus* | KF017148.1 |
| *Synodus foetens* | JN231001.1 |
| *Trachipterus arcticus* | KC442225.1 |
| *Trachurus trachurus* | EU638013.1 |
| *Tripterygion delaisi* | EU638016.1 |
| *Umbra limi* | JN230999.1 |
| *Valenciennea strigata* | EU638017.1 |
| *Vinciguerria nimbaria* | JN412570.1 |
| *Xiphias gladius* | EU638019.1 |
| *Xyrichtys novacula* | EU638020.1 |
| *Zeus faber* | EU638023.1 |
