# Supplementary material for: Functional trade-offs and environmental variation shaped ancient trajectories in the evolution of dim-light vision
Source: eLife. 2018 Oct 26;7:e35957. doi: 10.7554/eLife.35957 (PMC6203435; doi:10.7554/eLife.35957)
Supplement: Supplementary file 3. [file elife-35957-supp3.docx]

**Supplementary File 3**

| **Species** | ***Rh1* Accession Number** |
| --- | --- |
| *Apteronotus leptorhynchus* | KX260616.1 |
| *Ariopsis felis* | JN230993.1 |
| *Bagarius yarrelli* | JQ026296.1 |
| *Brachyhypopomus gauderio* | JX470074.1 |
| *Brycinus macrolepidotus* | JX470075.1 |
| *Chalceus macrolepidotus* | EU409633.1 |
| *Characidium fasciatum* | JX470076.1 |
| *Citharinus sp.* | JN230986.1 |
| *Clarias gariepinus* | JX470077.1 |
| *Cranoglanis bouderius* | JQ026294.1 |
| *Creteuchiloglanis kamengensis* | JQ026307.1 |
| *Ctenolucius hujeta* | JN230987.1 |
| *Danio rerio* | NM_131084.1 |
| *Dionda ipni voucher UAIC* | EU082607.1 |
| *Distichodus antonii* | JN230985.1 |
| *Eigenmannia virescens* | KX260614.1 |
| *Ellopostoma mystax* | FJ650477.1 |
| *Euchiloglanis kishinouyei* | JQ026301.1 |
| *Exostoma labiatum* | JQ026306.1 |
| *Gagata dolichonema* | JQ026297.1 |
| *Glaridoglanis andersonii* | JQ026303.1 |
| *Glyptothorax trilineatus* | JQ026298.1 |
| *Gymnotus cylindricus* | JX470078.1 |
| *Hepsetus odoe* | JX470079.1 |
| *Hoplias malabaricus* | JX470080.1 |
| *Hydrolycus armatus* | JX470081.1 |
| *Kryptopterus minor* | JN230992.1 |
| *Lacantunia enigmatica* | JX470082.1 |
| *Lefua costata* | EU409634.1 |
| *Leiarius pictus* | JX470083.1 |
| *Malapterurus microstoma* | JX470084.1 |
| *Microglanis iheringi* | JX470085.1 |
| *Nannostomus beckfordi* | JN230988.1 |
| *Oreoglanis macropterus* | JQ026304.1 |
| *Pangasianodon hypophthalmus* | JX255590.1 |
| *Pangio oblonga* | FJ197041.1 |
| *Parailia pellucida* | JX470086.1 |
| *Pareuchiloglanis gracilicaudata* | JQ026302.1 |
| *Phenacogrammus interruptus* | FJ197073.1 |
| *Pseudecheneis sulcata* | JQ026299.1 |
| *Pseudexostoma yunnanensis* | JQ026305.1 |
| *Rhamphichthys marmoratus* | JX470087.1 |
| *Rhinichthys cobitis* | JX443099.1 |
| *Schizodon cf. fasciatus* | JX470088.1 |
| *Semaprochilodus insignis* | JX470089.1 |
| *Silurus meridionalis* | JX255589.1 |
| *Steatogenys elegans* | JX470090.1 |
| *Sternopygus macrurus* | KX077603.1 |
